# Preferences for Meat and Meat Alternatives in Different Meal Scenarios: A Dutch Discrete Choice Experiment

**DOI:** 10.1016/j.cdnut.2026.109520

**Published:** 2026-08-26

**Authors:** Sander Biesbroek, Cora Klockenbusch, Jorien Veldwijk, Hans Dagevos, Maartje P Poelman

**Affiliations:** 1 Division of Human Nutrition and Health, Wageningen University & Research, Wageningen, The Netherlands; 2 Erasmus School of Health Policy & Management, Erasmus University Rotterdam, Rotterdam, The Netherlands; 3 Erasmus Choice Modelling Centre, Erasmus University Rotterdam, Rotterdam, The Netherlands; 4 Wageningen Social & Economic Research, Wageningen University & Research, Wageningen, The Netherlands; 5 Chair Group Consumption and Healthy Lifestyles, Wageningen University & Research, Wageningen, The Netherlands

**Keywords:** meat replacers, legumes, environmental sustainability, consumer preference, discrete choice experiment, sustainable diets

## Abstract

Understanding meat consumers’ food choice motives is needed to identify accelerating or limiting factors of the diet transition. This study investigated stated preferences for health, environmental, and production-related attributes when selecting meat or alternatives. A discrete choice experiment was conducted among 272 Dutch participants. Participants selected a meat or alternative ingredient for 2 meal scenarios: a Pasta and traditional Dutch potatoes–meat–vegetables (PMV) meal. In each scenario, participants completed 10 choice tasks in which they chose between meat, meat replacer, and legumes. Product attribute levels varied across tasks and included protein content, overall health score, overall environmental impact score, price, local production, and organic production. In both meal scenarios, participants preferred meat over the alternatives (β Pasta −0.51; β PMV −0.44) and legumes (β Pasta −0.91; β PMV −0.99). Across both scenarios, the health score, sustainability score, and price were consistently ranked as the most influential. Preference patterns differed across consumer subgroups. Approximately 30% of participants showed a particularly low willingness to pay for meat replacers and legumes, whereas other subgroups were considerably less negative toward these alternatives. Dutch consumers valued meat over meat replacers and legumes when planning for different meal types, despite ranking health and sustainability scores and price as the most important attributes. These findings confirm that providing health and sustainability information alone is insufficient to encourage a shift toward alternative protein sources and that additional strategies are needed to support dietary transitions.

## Introduction

Current food production and consumption in high-income countries harm both human and planetary health [1]. Meat, particularly red meat, is one of the main contributors to unhealthy and unsustainable diets [2,3]. As in many Western countries, mean meat consumption is higher than recommended in the Netherlands [4,5]. Therefore, substituting or partially replacing meat with protein-rich plant-based foods is one of the most efficient ways to improve the environmental and health impact of diets [6,7].

Protein-rich plant foods such as legumes, tofu, or seitan are only marginally consumed in Western countries, like the Netherlands [8]. Possible explanations are a lack of specific cooking skills, cultural factors, familiarity, or that these products have mostly been targeted at vegetarians or vegans without any explicit reference to their animal-based counterparts [[9], [10], [11]]. Plant-based meat alternatives (PBMAs) imitate the look, taste, specific use cases, and ways of preparing meat. Although these products could serve as a lower-threshold solution to replace meat, meat consumption remains the (social) norm [12,13]. Besides slight increases in vegan and vegetarian diet adherence, flexitarianism is emerging and represents a broad and heterogeneous dietary pattern characterized by reduced consumption of animal-based foods to varying degrees [4,14]. The gradual normalization of flexitarians is believed to support consumer demand for PBMAs because, at least at first glance, PBMAs seem to fit within the reduced-meat diet of flexitarians who seek palatable, nutritious, and affordable substitutes for conventional meat products [15]. Within the Dutch context, the range of PBMAs on the supermarket shelves has increased considerably in the last decade. However, in the Netherlands as well as in other countries, the market share of PBMAs remains relatively small compared with the overall market for meat products [16,17].

Although a slight reduction in meat consumption is noticeable, the shift is not yet substantial. There remains a limited understanding of where these changes occur and what motives drive them across different meal contexts. Understanding consumers’ food choice motives helps identify accelerating or limiting factors [12]. Predominant attributes in food choices are price, taste, health, and convenience [18]. However, many food items are not able to gratify all values at once, creating a dilemma in which consumers must prioritize or negotiate between values associated with different products [19]. In the Dutch context, characterized by an abundance of food and an overrepresentation of unhealthy and cheap food, consumers are not restricted by availability but rather consider another set of factors, such as time constraints, price, or cooking skills [18,19]. This prioritization may become increasingly difficult when factors, such as sustainability, animal welfare, and social justice, are added to the decision mix [20].

Food choice is influenced by a wide range of motivations, as reflected in comprehensive frameworks such as the Food Choice Questionnaire [21] and the Eating Motivation Survey [22]. These frameworks encompass diverse determinants of food choice, including price, health, and naturalness-related considerations, among many others. These frameworks have inspired the design of the choice tasks featured in the present study. Generally, consumers have previously been found to be willing to pay more for sustainable food products, including locally grown and organically produced options across a range of food categories [[23], [24], [25], [26]]. However, there is a notable shift in choices when it comes to meat and meat products. For example, expected taste tends to be valued over health and food safety when given the choice between meat products [27].

It is well established that food choices vary across contextual settings (e.g., supermarket, worksite canteen, or home) [28]. However, it remains unclear whether these contextual differences also occur between different meal types consumed at home. For example, eating behaviors may differ between a traditional Dutch potatoes–meat–vegetables (PMV) meal, in which the components are served separately, and commonly consumed pasta dishes, in which ingredients are typically mixed together. Gaining insight into how meat preferences differ across meal contexts is essential for designing effective interventions and communication strategies. Meals may evoke different traditions and sensory expectations, which influence people’s willingness to reduce or replace meat.

Although several consumer-oriented studies on the acceptance of PBMAs in the Netherlands have been published in recent years [[29], [30], [31], [32]], the impact of combining different domains of information (health, nutrition, costs, sustainability, and production), meal context, and the comparison of whole plant foods with meat alternatives on consumers’ choices remains unknown. Therefore, this exploratory study aims to identify Dutch consumers’ attitudes and preferences regarding different attributes (protein content, overall health, overall environmental impact, price, locally produced, and organically produced) of meat products, plant-based meat replacers, and legumes across different meal scenarios.

## Methods

### Study outline and recruitment

**FIGURE 1:**
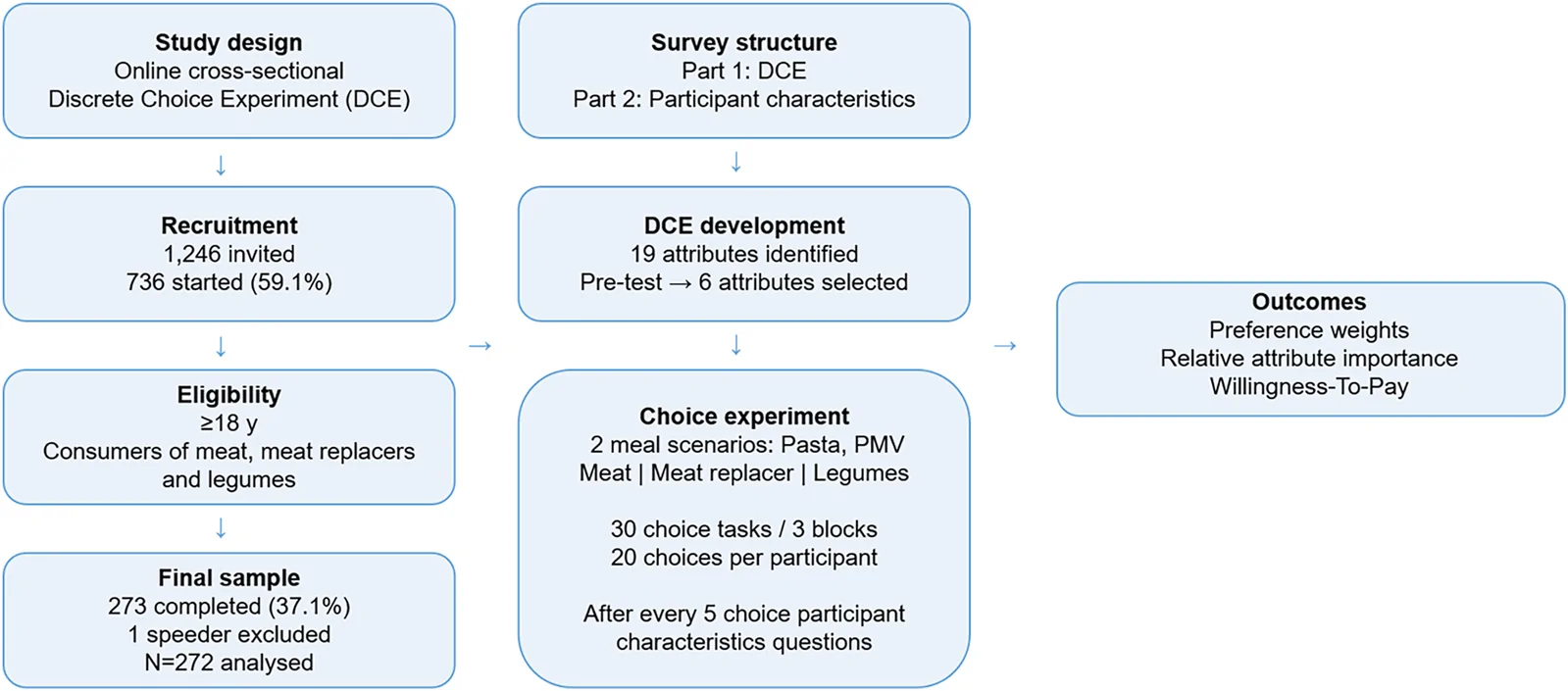
Flowchart and design of the experiment. PMV, potatoes–meat–vegetables.

A discrete choice experiment (DCE) was conducted to estimate the consumers’ willingness to pay (WTP) and the relative importance of different food attributes. The survey was an online cross-sectional design via Flycatcher Internet Research [33]. The full survey consisted of 2 parts, namely the DCE and questions on participants’ characteristics. An overview of the study design is shown in Figure 1.

The study aimed to recruit a sample that was broadly representative of the Dutch adult population with respect to age, sex, education, and region. Participants were recruited via the Flycatcher panel [33] in January 2022. At this time, the panel had 10,000 members in the Netherlands, applying a double-active-opt-in approach, and a voluntary participation with points as an incentive. Participants aged ≥18 y and who had consumed meat, meat replacers, and legumes in the past 3 mo were included. This also means that vegetarians and vegans were excluded from the study. The initial representative sample (based on quota sampling for gender, age, education, and region) that received an invitation to participate consisted of 1246 panelists, of whom 736 started with the inclusion questionnaire (response 59.1%). In total, 273 panelists met the inclusion criteria and completed the entire study (37.1%). The mean completion time was 905 s (∼15 min). One participant had a completion time below the standard Flycatcher cutoff [trimmed mean/3 = 241 s (∼4 min)] and was excluded. In total, 272 participants (37.0%) were included in the analyses.

### Discrete choice experiment

Before the choice tasks, participants were informed that there were no right or wrong answers and were encouraged to select the product they would most likely purchase in a real-life supermarket. Following this, 2 meal scenarios were used to represent different meal structures commonly found in Dutch cuisine. The respondents were asked to imagine buying ingredients for a specific meal. The first scenario was a pasta-based meal, in which meat is typically incorporated into the dish and mixed with other ingredients. The second scenario was a meal consisting of PMV, a common dinner combination in the Netherlands. In that scenario, the meat component is typically presented as a distinct and visible element on the plate, such as a steak or fillet.

A DCE is a stated-preference technique used to elicit individuals’ preferences and to quantify the relative importance of, for example, food products or service attributes [[34], [35], [36]]. In DCEs, respondents are asked to choose their preferred option from a set of alternatives, with choices typically based on hypothetical scenarios in stated-preference studies instead of revealing their choice in a real-world setting (e.g., a supermarket). Each product in a choice task, in this case a food product, is characterized by varying levels of attributes describing the product (e.g., in terms of price, sustainability, and health). It is assumed that the participant’s choice is formed based on the value or utility that the person assigns to the given attribute levels [37]. This allows researchers to assess an individual’s response to changes in attribute levels and estimate the utility associated with each attribute, from which the consumer’s WTP for an attribute of interest or market shares of products can be calculated [37].

#### Food product options

**FIGURE 2:**
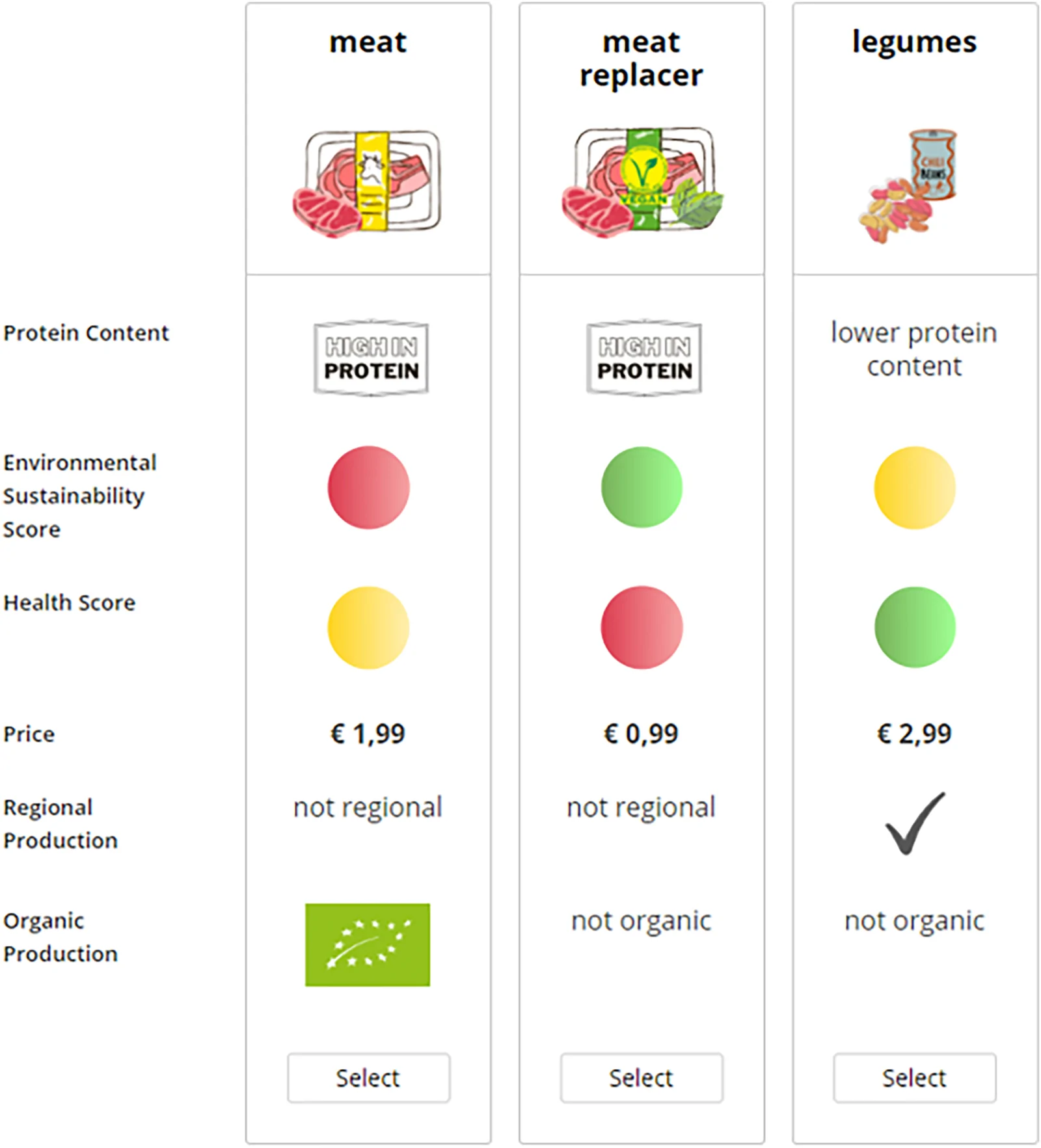
Example choice task from the pilot study conducted with international students. The final study employed the same questionnaire structure and design, administered in Dutch.

In this labeled DCE, participants were asked to choose between 3 options: meat, meat replacer, and legumes (Figure 2). An example of the Dutch questionnaire is available as Supplemental File 1. The 3 categories represent commonly consumed protein sources that can be integrated into a dinner meal. In the choice sets, they were displayed as broad food categories instead of concrete products to account for between-participant differences in familiarity and habitual consumption patterns. Using broader product categories rather than specific products allowed us to assess preferences for protein-source categories as they are encountered in everyday food choices. Examples of possible products for each category were listed in the scenario description before the choice tasks started. This was intended to help participants consider products that matched their own experiences, thereby creating a more realistic shopping situation.

#### Selection of attributes

An inventory of 19 product attributes for meat, meat analogs, and meat alternatives was compiled from the literature (Supplemental File 2). Because including such a high number of variables would increase respondent burden [38,39], a pretest was conducted to select 6 attributes. In this study, students ranked the 19 attributes in order of perceived importance, yielding a selection that largely corresponded with our proposed domain-based priority list. Six attributes were identified as the most relevant and were selected to cover both nutritional and health-related aspects (protein content and overall health) and various aspects of sustainability and production characteristics (overall sustainability label, production type, and production location) in a balanced manner (Table 1) [38,39]. Each attribute was explained to the participants in text before the online DCE task. The attribute levels were designed as simplified versions of familiar traffic light labeling systems and simplified statements such as the health claim “high in protein,” to facilitate rapid and intuitive interpretation of the information during the choice task [40].

**TABLE 1:** Selected attributes with associated levels

Attribute	Levels
Price	€0.99, €1.99, €2.99
Protein content	High-protein content, no label
General health label	Green, yellow, red
General sustainability	Green, yellow, red
Regional production	Yes, No
Organic production	Yes, No

#### Choice tasks

All 3 options (meat, meat replacer, and legumes) were displayed simultaneously (Figure 2). The order of the attributes was designed to minimize position effects and prevent the emergence of a perceived hierarchical order. Accordingly, the price attribute was positioned away from the top and bottom of the list, which are typically the most noticeable spots [41].

#### Participant characteristics

For behavioral information, participants were asked to indicate how often they consume products from the 3 categories and how they rated their overall taste. Further constructs were assessed, namely, a shortened Food Neophobia Scale [42], questions measuring meat attachment [43], a shortened Health Conscious Scale [44], and self-assessed knowledge of the different attributes using a 5-point Likert scale. Questions concerning characteristics of the study sample were mixed within the online survey and interrupted the choice sets after every 5 tasks. This was intended to make the survey as appealing as possible and reduce the burden of answering the choice tasks. The survey was generated and hosted on Sawtooth [45]. General participant characteristics assessed included age, gender, highest attained level of education, household size, net household income, religion, region, and type of work.

#### Experimental design

Using the Ngene software, a D-efficient design of 30 choice sets divided into 3 blocks was generated for the labeled DCE. Participants were asked to choose 10 times per scenario (Pasta and PMV). Respondents were randomly assigned to the blocks. Consequently, each participant was confronted with a total of 20 choice tasks. Respondents had unlimited time to choose. The design had 2 restrictions intended to enhance the plausibility of the choice scenarios to increase similarity to a real-world situation: it was preset not to allow the meat category to have a green environmental sustainability label, and the legumes category always showed the green health score.

#### Analyses

First, the responses were analyzed using a multinomial logit (MNL) model, which estimates the utility participants assign to each attribute level and to each alternative-specific constant (meat, meat replacer, or legumes). This analysis was conducted using the “Apollo” package in R (RStudio 2021.09.0). As utilities (regression coefficients) are influenced by a latent scale factor, they cannot be compared directly across classes. Instead, comparison was performed in the WTP space, in which estimates are comparable across classes. For this, each attribute regression parameter in the utility space was divided by the parameter for price [35].

Second, a latent class analysis (LCA) was conducted for both the Pasta and PMV scenarios to investigate heterogeneity in preferences. It assumes the presence of classes in the data with similar preferences. The LCA was performed using the lclogit2 and lclogitml2 functions in Stata 17, including a membership statement to estimate class probabilities of several baseline characteristics [46]. The number of classes retained in each scenario was determined by balancing improvements in model fit against the explanatory contribution of additional classes.

To rank the importance of the attributes, in the MNL model as well as the LCA, the difference between the highest and lowest attribute level estimate was calculated in the utility space. The largest difference value received an importance score of 1; the least important attribute received a score of 6.

## Results

### Participants

Of the total of 272 participants, 52.7% were female, and the mean age of the sample population was 47.5 y (Table 2). Most participants were highly educated (52.6%) and nonreligious (64.7%). Among the geographical regions, the western and most urbanized region in the Netherlands was the most represented (37.5%). More than half of the participants (56.3%) had a household net income between €20,000 and €60,000 per year, and the largest proportion of households (40.4%) consisted of 2 persons. Almost half of the participants indicated that they consumed meat 3 to 6 times per week (45.2%), meat replacers 1 to 2 times per week (44.5%), and legumes 1 to 2 times per week (51.8%) (Table 3). Furthermore, 77.9% of the participants ranked meat the tastiest option, compared with only 8.1% for legumes. Consistent with the quota sampling strategy, the sample was broadly representative of the Dutch adult population with regard to sex, age, and region [47]. Highly educated individuals remained somewhat overrepresented relative to national estimates, in which higher education is attained by about a third of the population [48].

**TABLE 2:** Descriptive statistics sample (*n* = 272)^a^

Characteristic	Sample	Characteristic	Sample
Females	143 (52.7)	Household size	
Age category, y		1	58 (21.3)
<30	53 (19.4)	2	120 (40.4)
30–50	85 (32.3)	3–4	80 (29.4)
50–65	87 (32.0)	>5	14 (5.2)
>65	47 (17.3)	Household net yearly income	
Education^b^		<₠20,000	34 (12.5)
Low	19 (7.0)	₠20,000–₠40,000	88 (32.4)
Middle	110 (40.4)	₠40,000–₠60,000	65 (23.9)
High	143 (52.6)	₠60,000–₠80,000	52 (19.1)
Religion		₠80,000–₠100,000	18 (6.6)
Nonreligious	176 (64.7)	>₠100,000	15 (5.5)
Christian	80 (29.4)	Work	
Other	16 (5.9)	Working	182 (66.9)
Region^c^		Unemployed	23 (8.5)
North	27 (9.9)	Student	21 (7.7)
Central	33 (12.1)	Retired	46 (16.9)
South	38 (14.0)		
West	102 (37.5)		
East	63 (23.2)		
South-West	9 (3.3)		

a Categorical variables are presented as frequencies (percentages).

b Education level was classified as low (lower vocational training or primary school), medium (intermediate vocational training or secondary school), or high (higher vocational training or university).

c North: Groningen, Friesland, and Drenthe; Central: Flevoland and Utrecht; South: Noord Brabant and Limburg; West: Noord and Zuid Holland; East: Gelderland and Overijssel; South-West: Zeeland.

**TABLE 3:** Current dietary behavior and attitudes (*n* = 272)^a^

Characteristic	Sample	Characteristic	Sample
Consumption frequencies		Ranking taste highest	
Meat^b^		meat taste	212 (77.9%)
<1 per 2 wk	32 (11.8%)	meat replacers’ taste	38 (14.0%)
1–2× per wk	107 (39.3%)	legumes’ taste	22 (8.1%)
3–6× per wk	123 (45.2%)	Food behavior concepts	
Every day	10 (3.7%)	Meat attachment scale^c^	2.73 (0.41)
Meat replacers		Health consciousness scale^d^	4.12 (0.55)
< 1 per 2 wk	117 (43.0%)	Food neophobia scale^e^	3.04 (0.36)
1–2× per wk	121 (44.5%)	Knowledge about the attributes^f^	
3–6× per wk	34 (12.5%)	Environmental impact	2.19 (0.68)
Legumes		Nutrition status and health	2.74 (0.65)
< 1 per 2 wk	109 (40.1%)	Macronutrients and nutritional value	2.15 (0.82)
1–2× per wk	141 (51.8%)	Food production practices	1.97 (0.73)
3–6× per wk	22 (8.1%)		

a Categorical variables are presented as frequencies (percentage), continuous variables as mean (SD).

b Meat includes all types of meat (beef, pork, and poultry) but not fish.

c Average score of 5 questions on meat attachment, all on a 5-point scale.

d Average score of 5 questions on health consciousness, all on a 5-point scale.

e Average score of 5 questions on food neophobia attachment, all on a 5-point scale.

f Reflects how the respondents rank their knowledge on several attributes, all on a 5-point scale.

### Preferences

In both the Pasta and PMV scenarios, participants reported an a priori preference of meat over meat replacers (β Pasta −0.51; β PMV −0.44) and legumes (β Pasta −0.91; β PMV −0.99) (Table 4). This indicates that, with all attributes being equal, participants would choose meat first, meat replacers second, and legumes last. In both scenarios, the attribute with the highest relative importance based on its utility rank was the health label (rank 1), followed by the sustainability label (rank 2). Price ranked third and fourth in the Pasta and PMV scenarios, respectively. The least important attributes in both scenarios were the protein label (rank 5) and the region production label (rank 6).

**TABLE 4:** Estimated attribute utility per scenario (*n* = 272)

Pasta scenario	Response coefficient	SE	Rank
High in protein^a^	0.23	0.05	5
Orange sustainability label^b^	0.55	0.07	—
Green sustainability label^b^	0.91	0.08	2
Orange health label^c^	0.74	0.08	—
Green health label^c^	1.17	0.07	1
Price	–0.47	0.03	3
Regional produced^d^	0.16	0.05	6
Organically produced^e^	0.45	0.05	4
Base utility meat replacer^f^	–0.51	0.06	—
Base utility legumes^f^	–0.91	0.07	—

PMV scenario	Response coefficient	SE	

High in protein^a^	0.18	0.05	5
Orange sustainability label^b^	0.72	0.07	—
Green sustainability label^b^	1.02	0.08	2
Orange health label^c^	0.83	0.08	—
Green health label^c^	1.30	0.08	1
Price	–0.43	0.03	4
Regional produced^d^	0.13	0.05	6
Organically produced^e^	0.53	0.05	3
Base utility meat replacer^f^	–0.44	0.06	—
Base utility legumes^f^	–0.96	0.07	—

Abbreviation: PMV, potatoes–meat–vegetables.

a Reference is lower in protein.

b Reference is a red environmental sustainability score.

c Reference is a red health score.

d Reference is not regionally produced.

e Reference is not organically produced.

f Reference is base utility meat.

To address potential preference heterogeneity, 3 latent classes were identified for both the Pasta and PMV meal scenarios (Table 5). For all attributes, the WTP estimates are presented; the full report of the LCA models is provided in Supplemental File 3.

**TABLE 5:** Class probabilities, WTP, and rank of attributes of the derived classes per scenario (*n* = 272)

Pasta scenario	Class 1 (0.37^a^)		Class 2 (0.27^a^)		Class 3 (0.36^a^)	
	WTP (95% CI)	Rank	WTP (95% CI)	Rank	WTP (95% CI)	Rank
High in protein^b^	0.99 (0.33, 1.65)	6	0.28 (–0.39, 0.95)	4	0.44 (0.25, 0.63)	5
Orange sustainability label^c^	2.67 (1.42, 3.92)	—	0.99 (–0.02, 2.00)	—	0.64 (0.31, 0.97)	—
Green sustainability label^c^	4.96 (2.98, 6.94)	2	1.27 (–0.04, 2.59)	2	0.90 (0.49, 1.32)	3
Orange health label^d^	4.25 (2.51, 5.98)	—	1.64 (0.66, 2.62)	—	0.76 (0.39, 1.13)	—
Green health label^d^	5.96 (3.71, 8.20)	1	2.18 (1.02, 3.33)	1	1.34 (0.90, 1.78)	1
Regional produced^e^	1.20 (0.45, 1.95)	4	0.25 (–0.45, 0.95)	5	0.23 (0.04, 0.42)	6
Organically produced^f^	2.66 (1.50, 3.82)	3	–0.01 (–0.63, 0.61)	6	0.61 (0.41, 0.80)	4
Price	—	5	—	3	—	2

PMV scenario	Class 1 (0.51^a^)		Class 2 (0.19^a^)		Class 3 (0.30^a^)	
	WTP (95% CI)	Rank	WTP (95% CI)	Rank	WTP (95% CI)	Rank
High in protein^b^	0.85 (0.43, 1.26)	6	0.11 (–0.09, 0.32)	5	0.21 (–1.13, 0.71)	6
Orange sustainability label^c^	2.20 (1.45, 2.94)	—	0.56 (0.32, 0.81)	—	3.17 (0.94, 5.41)	—
Green sustainability label^c^	3.93 (2.91, 4.95)	2	0.54 (0.20, 0.88)	3	4.48 (1.20, 7.77)	1
Orange health label^d^	3.78 (2.62, 4.94)	—	0.54 (0.24, 0.84)	—	3.09 (1.07, 5.10)	—
Green health label^d^	5.93 (4.34, 7.52)	1	0.64 (0.30, 0.99)	2	3.97 (1.51, 6.44)	2
Regional produced^e^	0.96 (0.51, 1.40)	5	0.01 (–0.23, 0.24)	6	0.49 (–0.53, 1.51)	5
Organically produced^f^	1.88 (1.33, 2.43)	3	0.41 (0.20, 0.61)	4	1.49 (0.31, 2.67)	3
Price	—	4	—	1	—	4

Abbreviations: CI, confidence interval; LCA, latent class analysis; PMV, potatoes–meat–vegetables; WTP, willingness to pay.

a Class probability of LCA.

b Reference is lower in protein.

c Reference is a red environmental sustainability score.

d Reference is a red health score.

e Reference is not regionally produced.

f Reference is not organically produced.

#### Pasta meal-type scenario

In the context of selecting a product for a pasta meal, data were distributed across 3 latent classes with the following probabilities: 37% for class 1, 27% for class 2, and 36% for class 3 (Table 5). Sociodemographic characteristics were associated with likely class membership (Supplemental Table 3.2). Male participants and younger respondents were more likely to belong to class 3. In contrast, participants aged >50 y were more likely to belong to class 2, followed by class 1. A similar pattern was observed for income, with an income of >€40,000 being associated with a larger likelihood of belonging to class 2 and then class 1. Class 2 membership likelihood also increased for households with >2 members. Regarding employment status, participants in class 1 had the highest likelihood of being employed, and those in class 2 had the lowest.

By dividing each attribute utility coefficient by the price utility coefficient, all attribute utilities were expressed as WTP estimates. For class 1, the base WTP was higher for meat replacers (€1.90) and legumes (€1.29) compared with meat. In class 2, the base WTP for the alternatives was lower than for meat: €−2.87 for meat replacers and €−11.02 for legumes. Class 3 had WTP estimates of €−0.83 and €−0.95 in favor of meat. In all 3 classes, health was the most important attribute, but WTP for a green compared with a red health label ranged from €5.96 in class 1 to €1.34 in class 3. The sustainability label ranked second in classes 1 and 2, whereas price was the second most important attribute in class 3. Price was only ranked fifth in class 1 and third in class 2. Protein content and regional production were not among the top 3 attributes in any class.

#### PMV meal-type scenario

In the PMV scenario, respondents had a probability of 0.51 to belong to class 1, 0.19 to belong to class 2, and 0.30 to belong to class 3 in the PMV scenario (Table 5). The likelihood of being nonreligious, having a lower education level, living in a smaller household, and having a yearly income of <€40,000 was lower in class 3 than in classes 1 and 2 (Supplemental Table 3.4). Participants in class 2 were more likely to be aged <50 y, male, and living outside the western part of the Netherlands compared with class 3. In addition, people in class 1 were more often older and more likely to live in the western part of the Netherlands.

In both classes 1 and 2, the base WTP was on average lower for meat replacers (€−0.29) and legumes (€−0.61) compared with meat. However, class 3 had lower WTP values with €−4.35 for meat replacers and €−15.76 for legumes. Classes 1 and 3 valued health (first in class 1 and second in class 3), sustainability (second in class 1 and first in class 3), and organic production most, compared with price, health, and sustainability in class 2. For all attributes, the WTP in this price-sensitive class 2 ranged between €0.01 for regionally produced products and €0.64 for a green health label, confirming a focus on price. In comparison, the WTP range in class 1 was €0.85 for a high-protein product to €5.93 for a green health label.

## Discussion

This experimental study evaluated Dutch consumer preferences for meat, plant-based meat replacers, and legumes with varying levels of 6 attributes in the context of 2 meal scenarios. By requiring respondents to make repeated trade-offs between sometimes competing product attributes, this study provides insights into behavioral preferences rather than isolated self-reported opinions. In these meals, a Pasta or a PMV dish, the attributes for health and sustainability (both traffic light labels) were ranked highest, and protein content and whether the food was regionally produced were ranked lowest in the total population sample. However, in the derived latent classes, considerable differences in attribute prioritization, ranking of the food alternatives, and WTP per attribute were observed, and these differed between meal scenarios. In general, price was a more important factor in the Pasta scenario compared with the PMV scenario. Only 1 class (class 1, probability of 0.37) in the Pasta scenario showed a positive WTP for meat replacers (€1.90) and legumes (€1,29). This highlights that, all attributes being equal, most of the participants in this survey would choose meat over the alternatives. Both scenarios showed a class with a very negative WTP for meat replacers and especially legumes, which was more pronounced in the PMV compared with the Pasta scenario. Interestingly, all attributes except for price had a mean positive utility coefficient in all scenarios and classes.

A recent Belgian choice experiment also examined the importance of different types of PBMA, meal context, and participant characteristics [25]. Although our study included legumes, Coucke et al. [25] investigated preferences for and place of hybrid (containing both animal and plant sources) and cultivated replacers. They found that plant-based meat analogs were preferred over hybrid plant-based meat analogs and that 60% of meat analog preference was predicted by meal context (masked, processed, hot, or cold serving) and consumption context (snack or meal). We observed only limited differences in WTP and latent class structure between the 2 meal scenarios. Similarly, Coucke et al. [25] concluded that meal type itself had only a limited impact on food choices.

In our study, a lower price was consistently favored. Although often ranked among the 3 most important attributes, cost was the most important determinant in only 1 latent class in the PMV scenario. This is not fully consistent with other findings, which often show price as the clear dominant determinant of grocery selection [49,50]. There is a difference, however, in the level of income and the importance of food prices and food affordability compared with the other latent classes. In addition, this DCE was a theoretical shopping experiment and thereby may have slightly reduced the importance of price. In our study, all attributes had positive WTP point estimates, indicating that for most attributes used in this study, a higher level (better performance/score) would justify a higher price for consumers. This might be a consequence of the experiment not being in a real-life setting but online, and the estimated WTP values may not reflect actual acceptable prices but rather reveal the ranking and order of magnitude of importance of the attributes themselves. When shopping, consumers purchase not only these specific foods. Therefore, the whole-food basket should be considered to address diet affordability [51]. The importance of price in reducing meat consumption was highlighted in a recent experiment in which meat purchases in supermarkets decreased the most when prices were increased by 30% compared with providing only information [52]. Interestingly, a previous study found that participants were willing to pay a premium for a healthier lasagne and also to pay a premium for lasagne with a lower carbon footprint, but this decreased when they knew these meals contained less meat [53]. This suggests strong feelings for keeping meat in the diet, even at the expense of sustainability. To facilitate the diet transition, it is key that foods beneficial for health and the environment are affordable for all. In addition, one could argue that unhealthy and environmentally unsustainable foods are now too cheap because their prices do not reflect their negative impacts on society, such as greenhouse gas emissions and health impacts, in the food price [54].

Using our DCE, we were able to examine how various attributes from multiple domains influence preferences across distinct meal contexts. Although not directly comparable because of scaling, the smaller negative WTP estimates for legumes and meat replacers in the Pasta compared with the PMV scenario might indicate that mixed meals offer a better opportunity to increase consumer acceptance of a lower meat content, meat substitution, and alternative protein sources. Differences in WTP for meat alternatives between the 2 meal contexts may be partly explained by the cultural and sensory meanings attached to specific meals (PMV/Pasta). In traditional PMV meals, meat holds a central and symbolic role as the defining component, which makes consumers less inclined to substitute it with alternatives [55]. In contrast, mixed meals like a pasta are often perceived as more flexible in composition, and meat is less essential for the recipe [56]. In another study on PBMA acceptance in Dutch adults, researchers observed diverse patterns among different dietary groups, but among dedicated flexitarians and vegetarians, there was an overall higher preference for PBMA in a PMV rather than a mixed-meal setting [57]. Our DCE clearly showed that most people do not perceive meat replacers and legumes as equally valuable alternatives to meat. This lower WTP might be less concerning for legumes, which are generally much cheaper, but many meat replacers are priced very similarly to meat. The added value of lower environmental impact or health should thus be made clearer for consumers to support informed purchasing decisions.

These analyses provide detailed insights into which attributes drive food decision-making in the supermarket and how context matters. During the choice tasks, the participants had access to all information in one visual overview to support decision-making. But even with this information available, the base utilities for meat replacers and legumes remained lower than those for meat. Providing additional information alone might not steer consumers away from meat purchases. In addition, information on all these attributes is, in practice, not available or at least not in a uniform format, which may contribute to consumer confusion [58]. These domains include product-specific attributes (protein content, nutritional quality) but also more system-oriented attributes (sustainability, production type, or animal welfare) and postconsumption effects (e.g., health outcomes), thereby further complicating choices. Although food products from local farmers or produced organically receive much attention and are linked to labels suggesting greater sustainability, these attributes hardly impacted consumer choices in our experiment. Another dimension that has received increasing attention is the distinction between minimally processed whole foods and ultraprocessed foods [59,60]. This is relevant to the present study because legumes represent a whole-food alternative, whereas many plant-based meat replacers are classified as ultraprocessed foods. However, the plant-based meat replacer category is highly heterogeneous, and there remains substantial scientific debate regarding whether health outcomes are driven primarily by the degree of processing or by the nutritional composition of the food [61,62]. However, consumers’ general skepticism regarding technologies in the food domain remains a significant challenge [63].

Of the different attributes, health, sustainability, and price ranked consistently high in this sample. In contrast, Van Bussel et al. [64] found that consumers themselves do not perceive the environmental impact of food as a strong influence on their food choice, which aligns with findings from previous choice experiments [[65], [66], [67]] and related systematic reviews [68,69]. The low importance of the organic attribute in our study appears inconsistent with studies reporting growing consumer interest in organic foods [70]. Although price plays a key role in food choice, taste and sensory qualities must meet consumer expectations to encourage repeated purchases [71]. These sensory aspects could not be captured in the present experiment.

For consumers, it is often difficult to make informed choices with the sustainability of products in mind, partly because of the lack of standardized methods and labels [72,73]. Sustainability is a difficult domain to capture in a single number, label, or logo. Given the potential importance of sustainability in food choice in our experiment, efforts to develop standardized methods for life cycle assessment of food products to quantify these impacts and present them on labels should, although highly challenging to develop and validate, be a priority for policymakers and the food industry [[73], [74], [75], [76]]. Other, more directive options can include incorporating environmental and/or health externalities in food prices [12,54,77]. This way, price remains the leading determinant but more accurately reflects impacts on other dimensions, such as environmental sustainability.

In essence, in both meal-type scenarios, 30% of the population was unlikely to choose meat replacers or legumes over meat. On a positive note, the rest of the population displayed a small positive or small negative WTP toward the replacers. For these groups, providing attribute information through front-of-package labels might convince them to buy these foods more often. Class 1 in the Pasta scenario showed a positive WTP toward legumes and meat replacers. In this class, participants had a higher likelihood of being female, aged >50 y, nonreligious, having a higher income, and living in the western part of the Netherlands. These characteristics are comparable with profiles of flexitarians, vegetarians, and vegans in the literature [14,78,79]. Both meal-type scenarios also included a class—class 2 (membership probability of 0.27) in the Pasta and class 3 (membership probability of 0.30) in the PMV scenario, with a very low WTP for legumes and meat replacers compared with meat. In the PMV scenario, the likelihood of being from a larger household, having a higher level of education, and being religious was higher compared with the other classes in that scenario. Similarly, participants in the low WTP class in the Pasta scenario were more likely to come from a larger household and have a higher education. In contrast to the PMV scenario class, the likelihood of them being female, older, and living in the western part of the Netherlands was higher. One similarity between these 2 classes appear to be a higher likelihood of belonging to larger households and having a higher level of education. It might be possible that, with an increasing number of family members, it becomes more difficult to align the interests of all household members and facilitate and implement changes toward plant-based diets. Such suggestions are directly related to the growing scholarly interest in household and family dynamics in dietary choice and change, taking as its point of departure that food choices and dietary transitions are not only shaped by individual motivations but also by interpersonal relationships. Given that food choices and preferences are not made in isolation but are deeply and diversely socioculturally laden, it is highly relevant that, currently, a series of studies highlight that eating is a social activity and, accordingly, devote attention to how social interactions and networks influence dietary choice and change [[80], [81], [82]].

A choice experiment like this also comes with both strengths and limitations. The consumer panel allowed us to recruit a sample that was broadly representative of the Dutch population in terms of age, sex, education, and region. However, because participants needed to have consumed meat, meat replacers, and legumes in the past 3 mo to ensure familiarity with all product categories, this led to the exclusion of people who had, for example, never consumed meat replacers. For these people, the WTP for these products might be even lower, although for vegans and vegetarians (both excluded in this experiment), the base WTP would likely be higher. The 2 meal-based scenarios’ settings allowed us to differentiate between meal context and WTP for the alternative foods. We decided to use general food group names (meat, meat replacer, and legumes) instead of specific types of products. This allowed us to account for between-participant variation in taste and habitual consumption. However, this approach reduced the specificity of the scenarios because it remains unknown if all participants understood the experiment correctly and in the same way. The alternative would have been to show specific food products, such as minced meat, vegan minced meat, and lentils, but that could have introduced bias and heterogeneity related to the preferences for individual food products and influenced the results as well. Although the order of the choice tasks was randomized across participants, the repeated exposure to attribute information may have increased familiarity with the attributes or influenced subsequent decisions over time. Furthermore, taste expectations and habitual consumption patterns were not included in the DCE measurements. Both concepts were incorporated in the baseline questions, which may be useful for future analyses. The DCE measured stated preferences in a hypothetical online setting rather than actual purchasing behavior, and WTP estimates may therefore differ from behavior in real-world food environments. In addition, attribute combinations may have been perceived as less realistic by respondents, despite efforts to improve the plausibility of the scenarios.

In conclusion, in this stated-choice experiment in the Dutch adult population, most participants valued plant-based meat replacers and legumes less than meat in 2 meal scenarios, but mixed Pasta meals showed greater potential than traditional PMV meals for meat replacement. Of the proposed attributes, health, sustainability, and price ranked consistently highest. Providing participants with information on all attributes did not steer consumer choice away from meat, which confirms the frequently established finding that information provision alone is an insufficient intervention to effectively encourage a transition toward more healthy and sustainable food consumption.

## Author contributions

The authors’ responsibilities were as follows – SB: contributed to funding acquisition, conceptualization, methodology, formal analysis, and writing – original draft; CK: contributed to methodology, data curation, formal analysis, and writing – review and editing; JV: contributed to methodology and writing – review and editing; HD: contributed to funding acquisition and writing – review and editing; MPP: contributed to funding acquisition, conceptualization, supervision, and writing – review and editing; and all authors: read and approved the final version for submission.

## Data availability

Data described in the manuscript, code book, and analytic code can be made available on request pending application and approval.

## Funding

This project was part of the Dutch Science Agenda (NWA) program “Transition toward a sustainable food system,” funded by the Dutch Research Organisation (NWO): NWA.1235.18.201.

## Declaration of generative AI and AI-assisted technologies in the writing process

The authors declare that no generative AI or AI-assisted technologies were used in the writing of this manuscript.

## Conflict of interest

The authors report no conflicts of interest.
