# Supplementary material for: Preferences for Meat and Meat Alternatives in Different Meal Scenarios: A Dutch Discrete Choice Experiment
Source: Curr Dev Nutr. 2026 Aug 26;10(9):109520. doi: 10.1016/j.cdnut.2026.109520 (PMC13597198; doi:10.1016/j.cdnut.2026.109520)
Supplement: Supplementary file 1 [file mmc1.docx]

Supplementary file 1. **Outline of the experiment**

# **Preferences for meat and meat alternatives in different meal scenarios:** **a Dutch discrete choice experiment**

Sander Biesbroek^a^, Cora Klockenbusch^a^, Jorien Veldwijk^b,c^, Hans Dagevos^d^, Maartje P. Poelman^e^

========================================

Question List

========================================

Start (Questionnaire Access)

Intro (Text)

Consent (Select)

NoConsent (Terminate)

Intro2 (Text)

MeATGrid5 (Grid)

ConsumptionFrequencies3 (Grid)

TastePerception (Ranking)

DescriptionDCE (Text)

AttributeDescription (Text)

Scenario1Pasta (Text)

PastaDinner_Random1 (CBC Random Task)

PastaDinner_Random2 (CBC Random Task)

PastaDinner_Random3 (CBC Random Task)

PastaDinner_Random4 (CBC Random Task)

PastaDinner_Random5 (CBC Random Task)

Gender (Select)

Age (Numeric)

Religion (Select)

CulturalBackground (Select)

PastaDinner_Random6 (CBC Random Task)

PastaDinner_Random7 (CBC Random Task)

PastaDinner_Random8 (CBC Random Task)

PastaDinner_Random9 (CBC Random Task)

PastaDinner_Random10 (CBC Random Task)

AttriKnowledge (Grid)

TextScenario2 (Text)

AGV_Random1 (CBC Random Task)

AGV_Random2 (CBC Random Task)

AGV_Random3 (CBC Random Task)

AGV_Random4 (CBC Random Task)

AGV_Random5 (CBC Random Task)

Woonplaats (Select)

BurgerlijkeStatus (Select)

Huishoudgrootte (Select)

PersonalIncome (Select)

AGV_Random6 (CBC Random Task)

AGV_Random7 (CBC Random Task)

AGV_Random8 (CBC Random Task)

AGV_Random9 (CBC Random Task)

AGV_Random10 (CBC Random Task)

HealthConsciousness (Grid)

FoodNeophobia (Grid)

SubstitutionMeatfreeDays (Grid)

Werkstatus (Select)

HoogsteEdu (Select)

Terminate (Terminate)
