# Supplementary material for: Preferences for Meat and Meat Alternatives in Different Meal Scenarios: A Dutch Discrete Choice Experiment
Source: Curr Dev Nutr. 2026 Aug 26;10(9):109520. doi: 10.1016/j.cdnut.2026.109520 (PMC13597198; doi:10.1016/j.cdnut.2026.109520)
Supplement: Supplementary file 2 [file mmc2.docx]

Supplementary file 2. **Inventory of product attributes**

# **Preferences for meat and meat alternatives in different meal scenarios:** **a Dutch discrete choice experiment**

Sander Biesbroek^a^, Cora Klockenbusch^a^, Jorien Veldwijk^b,c^, Hans Dagevos^d^, Maartje P. Poelman^e^

**Supplemental Table A1.** Inventory of possible product attributes

| **Attribute** | **Level** | **Literature** |
| --- | --- | --- |
| **Product - 6** |  |  |
| Price | avr - 15% / average / avr + 15%; +/- 30% | Lambooij et al., 2019 |
| Source | animal / ovo-lacto / plant (by product choice) | - |
| Ingredients list | 1 / 2 - 4 / > 5 ingredients | Weinrich, 2018 |
| Portion Size | Single / two people / family (maybe in grams) | - |
| Time Needed for Preparation | < 5 min / 5 - 10 min / > 10 min  ready to cook / plain | Dudinskaya et al., 2021 |
| Animal Welfare |  | Gross et al., 2021 |
| Production | organic / conventional | Vanhonacker et al., 2013  Hasselbach & Roosen, 2015  Profeta et al., 2021 |
| **Health - 5** |  |  |
| Protein Content | high protein content / no label | Dudinskaya et al., 2021 |
| Health Label (general) | red / yellow / green | (DCEs with a lot of different health attributes) |
| Different Nutritional Factors | high content of unsaturated fatty acids / no label / high in fibre | Profeta et al., 2021 |
| Fat Content | low fat / fatty | Dudinskaya et al., 2021 |
| Food Safety | frozen / non-frozen | Lambooij et al., 2019 |
| **Sustainability - 5** |  |  |
| Sustainability Label | red / yellow / green | Very often included |
| Environmental Claims | [20% reduced carbon footprint] | Dudinskaya et al., 2021  Profeta et al., 2021 |
| Origin | distance travelled OR  regional / from neighbouring country / from far away OR  regional / no label | Profeta et al., 2021  Hasselbach & Roosen, 2015 |
| Livestock Production System | indoor / free range / organic  plants: conventional / organic | Lambooij et al., 2019 |

**References:**

Dudinskaya, E. C., Naspetti, S., Arsenos, G., Caramelle-Holtz, E., Latvala, T., Martin-Collado, D., Orsini, S., Ozturk, E., & Zanoli, R. (2021). European Consumers’ Willingness to Pay for Red Meat Labelling Attributes. Animals 2021, Vol. 11, Page 556, 11(2), 556. https://doi.org/10.3390/ANI11020556

Gross, S., Waldrop, M. E., & Roosen, J. (2021). How does animal welfare taste? Combining sensory and choice experiments to evaluate willingness to pay for animal welfare pork. Food Quality and Preference, 87, 104055. https://doi.org/10.1016/J.FOODQUAL.2020.104055

Hasselbach, J. L., & Roosen, J. (2015). Motivations behind Preferences for Local or Organic Food. Http://Dx.Doi.Org/10.1080/08961530.2015.1022921, 27(4), 295–306. https://doi.org/10.1080/08961530.2015.1022921

Lambooij, M. S., Veldwijk, J., van Gils, P., Mangen, M. J. J., Over, E., Suijkerbuijk, A., Polder, J., de Wit, G. A., & Opsteegh, M. (2019). Consumers’ preferences for freezing of meat to prevent toxoplasmosis– A stated preference approach. Meat Science, 149, 1–8. https://doi.org/10.1016/J.MEATSCI.2018.11.001

Profeta, A., Baune, M. C., Smetana, S., Broucke, K., van Royen, G., Weiss, J., Heinz, V., & Terjung, N. (2021). Discrete choice analysis of consumer preferences for meathybrids—findings from germany and belgium. Foods, 10(1). https://doi.org/10.3390/FOODS10010071

Vanhonacker, F., Loo, E. J. van, Gellynck, X., & Verbeke, W. (2012). Flemish consumer attitudes towards more sustainable food choices q. https://doi.org/10.1016/j.appet.2012.11.003

Weinrich, R. (2018). Cross-Cultural Comparison between German, French and Dutch Consumer Preferences for Meat Substitutes. Sustainability 2018, Vol. 10, Page 1819, 10(6), 1819. https://doi.org/10.3390/SU10061819
