# Supplementary material for: Preferences for Meat and Meat Alternatives in Different Meal Scenarios: A Dutch Discrete Choice Experiment
Source: Curr Dev Nutr. 2026 Aug 26;10(9):109520. doi: 10.1016/j.cdnut.2026.109520 (PMC13597198; doi:10.1016/j.cdnut.2026.109520)
Supplement: Supplementary file 3 [file mmc3.docx]

Supplementary file 3. **DCE full results**

# **Preferences for meat and meat alternatives in different meal scenarios:** **a Dutch discrete choice experiment**

Sander Biesbroek^a^, Cora Klockenbusch^a^, Jorien Veldwijk^b,c^, Hans Dagevos^d^, Maartje P. Poelman^e^

**Supplemental Table 3.1.** Class probabilities and utilities of the Pasta scenario (n=272).

| **Class 1** (0.37^a^) | Response Coefficient | Std err | z | P>z | [95%CI] | |
| --- | --- | --- | --- | --- | --- | --- |
| High in protein^b^ | 0.34 | 0.10 | 3.55 | 0.00 | 0.15 | 0.53 |
| Orange sustainability label^c^ | 0.91 | 0.16 | 5.66 | 0.00 | 0.60 | 1.23 |
| Green sustainability label^c^ | 1.70 | 0.18 | 9.57 | 0.00 | 1.35 | 2.04 |
| Orange health label^d^ | 1.15 | 0.20 | 7.15 | 0.00 | 1.05 | 1.85 |
| Green health label^d^ | 2.04 | 0.21 | 9.74 | 0.00 | 1.63 | 2.45 |
| Price | -0.34 | 0.07 | -5.22 | 0.00 | -0.47 | -0.21 |
| Regional produced^e^ | 0.41 | 0.10 | 3.95 | 0.00 | 0.21 | 0.61 |
| Organically produced^f^ | 0.91 | 0.11 | 8.35 | 0.00 | 0.70 | 1.12 |
| Base utility meat replacer^g^ | 0.65 | 0.22 | 2.98 | 0.00 | 0.22 | 1.08 |
| Base utility legumes^g^ | 0.44 | 0.23 | 1.92 | 0.06 | -0.01 | 0.89 |
|  |  |  |  |  |  |  |
| **Class 2** (0.27^a^) | Response Coefficient | Std err | z | P>z | [95%CI] | |
| High in protein^b^ | 0.10 | 0.12 | 0.84 | 0.40 | -0.13 | 0.32 |
| Orange sustainability label^c^ | 0.35 | 0.15 | 2.25 | 0.02 | 0.04 | 0.65 |
| Green sustainability label^c^ | 0.44 | 0.22 | 2.05 | 0.04 | 0.02 | 0.87 |
| Orange health label^d^ | 0.57 | 0.13 | 4.37 | 0.00 | 0.32 | 0.83 |
| Green health label^d^ | 0.76 | 0.13 | 5.70 | 0.00 | 0.50 | 1.02 |
| Price | -0.35 | 0.07 | -4.67 | 0.00 | -0.50 | -0.20 |
| Regional produced^e^ | 0.09 | 0.13 | 0.68 | 0.50 | -0.17 | 0.34 |
| Organically produced^f^ | 0.00 | 0.11 | -0.04 | 0.97 | -0.22 | 0.21 |
| Base utility meat replacer^g^ | -1.00 | 0.12 | -8.04 | 0.00 | -1.24 | -0.76 |
| Base utility legumes^g^ | -3.84 | 0.31 | -12.47 | 0.00 | -4.45 | -3.24 |
|  |  |  |  |  |  |  |
| **Class 3** (0.36^a^) | Response Coefficient | Std err | z | P>z | [95%CI] | |
| High in protein^b^ | 0.50 | 0.11 | 4.59 | 0.00 | 0.29 | 0.72 |
| Orange sustainability label^c^ | 0.74 | 0.18 | 4.13 | 0.00 | 0.39 | 1.09 |
| Green sustainability label^c^ | 1.04 | 0.22 | 4.66 | 0.00 | 0.60 | 1.48 |
| Orange health label^d^ | 0.87 | 0.20 | 4.37 | 0.00 | 0.48 | 1.27 |
| Green health label^d^ | 1.54 | 0.22 | 7.11 | 0.00 | 1.12 | 1.96 |
| Price | -1.15 | 0.09 | -12.70 | 0.00 | -1.33 | -0.97 |
| Regional produced^e^ | 0.27 | 0.11 | 2.43 | 0.02 | 0.05 | 0.48 |
| Organically produced^f^ | 0.70 | 0.11 | 6.64 | 0.00 | 0.49 | 0.90 |
| Base utility meat replacer^g^ | -0.96 | 0.17 | -5.76 | 0.00 | -1.29 | -0.63 |
| Base utility legumes^g^ | -1.09 | 0.17 | -6.49 | 0.00 | -1.42 | -0.76 |

a. Class probability of LCA

b. Reference is lower in protein

c. Reference is a red environmental sustainability score

d. Reference is a red health score

e. Reference is not regional

f. Reference is not organically produced

g. Reference is base utility meat

**Supplemental Table 3.2.** Class membership of the classes for the Pasta meal type (n=272).

| **Variable** | **Class 1** | **Class 2** | **Class 3** |
| --- | --- | --- | --- |
| Male | -0.63 | -0.42 | 0 |
| Age >50 years | 0.87 | 1.22 | 0 |
| Religious | -0.25 | -0.22 | 0 |
| High education^a^ | -0.09 | 0.13 | 0 |
| Household size >2 persons | -0.45 | 0.48 | 0 |
| Income above €40.000 | 0.57 | 0.70 | 0 |
| Other than Randstad^b^ | -0.63 | -0.46 | 0 |
| Working | 0.41 | -0.34 | 0 |

a. Higher vocational training or university.

b. Drenthe, Groningen, Friesland, Zeeland, Limburg, Noord Brabant, Gelderland, Overijssel

**Supplemental Table 3.3.** Class probabilities and utilities of the PMV scenario (n=272).

| **Class 1** (0.51^a^) | Response Coefficient | Std err | z | P>z | [95%CI] | |
| --- | --- | --- | --- | --- | --- | --- |
| High in protein^b^ | 0.36 | 0.08 | 4.57 | 0.00 | 0.21 | 0.51 |
| Orange sustainability label^c^ | 0.93 | 0.12 | 7.64 | 0.00 | 0.69 | 1.17 |
| Green sustainability label^c^ | 1.67 | 0.15 | 11.31 | 0.00 | 1.38 | 1.96 |
| Orange health label^d^ | 1.60 | 0.19 | 8.48 | 0.00 | 1.23 | 1.97 |
| Green health label^d^ | 2.52 | 0.21 | 12.01 | 0.00 | 2.11 | 2.93 |
| Price | -0.42 | 0.05 | -8.42 | 0.00 | -0.52 | -0.33 |
| Regional produced^e^ | 0.41 | 0.09 | 4.71 | 0.00 | 0.24 | 0.58 |
| Organically produced^f^ | 0.80 | 0.08 | 9.78 | 0.00 | 0.64 | 0.96 |
| Base utility meat replacer^g^ | -0.12 | 0.13 | -0.95 | 0.34 | -0.38 | 0.13 |
| Base utility legumes^g^ | -0.26 | 0.12 | -2.17 | 0.03 | -0.50 | -0.03 |
|  |  |  |  |  |  |  |
| **Class 2** (0.19^a^) | Response Coefficient | Std err | z | P>z | [95%CI] | |
| High in protein^b^ | 0.19 | 0.17 | 1.10 | 0.27 | -0.15 | 0.52 |
| Orange sustainability label^c^ | 0.93 | 0.21 | 4.50 | 0.00 | 0.53 | 1.34 |
| Green sustainability label^c^ | 0.89 | 0.29 | 3.07 | 0.00 | 0.32 | 1.46 |
| Orange health label^d^ | 0.89 | 0.25 | 3.58 | 0.00 | 0.40 | 1.38 |
| Green health label^d^ | 1.06 | 0.27 | 3.90 | 0.00 | 0.53 | 1.98 |
| Price | -1.65 | 0.18 | -9.38 | 0.00 | -2.00 | -1.31 |
| Regional produced^e^ | 0.01 | 0.20 | 0.05 | 0.96 | -0.37 | 0.39 |
| Organically produced^f^ | 0.67 | 0.16 | 4.11 | 0.00 | 0.35 | 0.99 |
| Base utility meat replacer^g^ | -0.26 | 0.20 | -1.28 | 0.20 | -0.65 | 0.14 |
| Base utility legumes^g^ | -0.93 | 0.30 | -3.12 | 0.00 | -1.52 | -0.35 |
|  |  |  |  |  |  |  |
| **Class 3** (0.30^a^) | Response Coefficient | Std err | z | P>z | [95%CI] | |
| High in protein^b^ | -0.05 | 0.11 | -0.45 | 0.65 | -0.26 | 0.16 |
| Orange sustainability label^c^ | 0.74 | 0.15 | 5.04 | 0.00 | 0.45 | 1.03 |
| Green sustainability label^c^ | 1.04 | 0.22 | 4.68 | 0.00 | 0.61 | 1.48 |
| Orange health label^d^ | 0.72 | 0.13 | 5.73 | 0.00 | 0.47 | 0.96 |
| Green health label^d^ | 0.93 | 0.13 | 6.92 | 0.00 | 0.66 | 1.19 |
| Price | -0.23 | 0.07 | -3.44 | 0.00 | -0.37 | -0.10 |
| Regional produced^e^ | 0.11 | 0.12 | 0.96 | 0.34 | -0.12 | 0.35 |
| Organically produced^f^ | 0.35 | 0.10 | 3.36 | 0.00 | 0.14 | 0.55 |
| Base utility meat replacer^g^ | -1.01 | 0.14 | -7.08 | 0.00 | -1.29 | -0.73 |
| Base utility legumes^g^ | -3.67 | 0.28 | -13.28 | 0.00 | -4.21 | -3.13 |

a. Class probability of LCA

b. Reference is lower in protein

c. Reference is a red environmental sustainability score

d. Reference is a red health score

e. Reference is not regional

f. Reference is not organically produced

g. Reference is base utility meat

**Supplemental Table 3.4.** Class membership of the classes in the PMV scenario (n=272).

| **Variable** | **Class 1** | **Class 2** | **Class 3** |
| --- | --- | --- | --- |
| Males | -0.11 | 0.17 | 0 |
| Age >50 year | 0.10 | -0.55 | 0 |
| Religious | -0.09 | -0.18 | 0 |
| High education^a^ | -0.39 | -0.41 | 0 |
| Household size >2 persons | -0.42 | -0.69 | 0 |
| Income above €40,000 | -0.30 | -0.39 | 0 |
| Other than Randstad^b^ | -0.17 | 0.29 | 0 |
| Working | 0.58 | -0.80 | 0 |

a. Higher vocational training or university.

b. Drenthe, Groningen, Friesland, Zeeland, Limburg, Noord Brabant, Gelderland, Overijssel
